# Pharmacotherapy Treatment Options for Insomnia: A Primer for Clinicians

**DOI:** 10.3390/ijms17010050

**Published:** 2015-12-30

**Authors:** Gregory M. Asnis, Manju Thomas, Margaret A. Henderson

**Affiliations:** 1 Albert Einstein College of Medicine/Montefiore Medical Center, Department of Psychiatry & Behavioral Science, Bronx, NY 10467, USA; 2 The Anxiety and Depression Clinic, Bronx, NY 10570, USA; manju.thomas3@gmail.com (M.T.); mahg96@aol.com (M.A.H.)

**Keywords:** insomnia, hypnotics, treatment options, guidelines

## Abstract

Insomnia is a prevalent disorder with deleterious effects such as decreased quality of life, and a predisposition to a number of psychiatric disorders. Fortunately, numerous approved hypnotic treatments are available. This report reviews the state of the art of pharmacotherapy with a reference to cognitive behavioral therapy for insomnia (CBT-I) as well. It provides the clinician with a guide to all the Food and Drug Administration (FDA) approved hypnotics (benzodiazepines, nonbenzodiazepines, ramelteon, low dose sinequan, and suvorexant) including potential side effects. Frequently, chronic insomnia lasts longer than 2 years. Cognizant of this and as a result of longer-term studies, the FDA has approved all hypnotics since 2005 without restricting the duration of use. Our manuscript also reviews off-label hypnotics (sedating antidepressants, atypical antipsychotics, anticonvulsants and antihistamines) which in reality, are more often prescribed than approved hypnotics. The choice of which hypnotic to choose is discussed partially being based on which segment of sleep is disturbed and whether co-morbid illnesses exist. Lastly, we discuss recent label changes required by the FDA inserting a warning about “sleep-related complex behaviors”, e.g., sleep-driving for all hypnotics. In addition, we discuss FDA mandated dose reductions for most zolpidem preparations in women due to high zolpidem levels in the morning hours potentially causing daytime carry-over effects.

## 1. Treatment Options for Insomnia: A Primer for Clinicians

Chronic insomnia is a prevalent (10%) medical disorder defined by difficulty falling or staying asleep or non-restorative sleep combined with impaired daytime functioning [1]. It is associated with increased motor vehicle accidents, and falls [2], increased healthcare utilization [3], and decreased survival rates [4]. Insomnia predisposes to the development of a number of psychiatric disorders, particularly depressive and anxiety disorders [5,6]. When psychiatric disorders are present, insomnia is associated with greater severity of illness including suicidal behavior [7,8,9]. Successful treatment of insomnia frequently leads to an earlier antidepressant and anxiolytic response [10,11]. Insomnia also interacts with various medical conditions; it can predispose to the development of type 2 diabetes, metabolic syndrome, and obesity [12]. In addition, treatment of insomnia co-morbid with a medical illness may lead to improvement of both conditions [13]. Thus, there is a clear need to treat insomnia.

This paper explores what alternatives (and their side effects) clinicians have available to treat insomnia, focusing predominantly on pharmacotherapy, and what situations warrant one treatment over another.

The two most widely accepted treatments for insomnia are hypnotic medications and cognitive behavioral therapy for insomnia (CBT-I) [1]. The latter is a structured treatment utilizing behavioral strategies including relaxation techniques, sleep hygiene, stimulus control, sleep restriction and cognitive techniques. These methods spotlight negative and distorted cognitions and behaviors associated with insomnia. CBT-I is administered over a 5 h period (4 to 6 weeks) and can be administered monthly (maintenance treatment) [14,15].

Hypnotic medications act quickly, usually after the first dose in contrast to CBT-I which takes weeks. Thus, many prefer medication. Few studies have compared hypnotic medications directly to CBT-I; short-term outcomes were similar. After discontinuing treatments, CBT patients did better than the medication group for periods assessed up to 1 year [1,14,15]; thus, there seems to be a learned carry-over effect for CBT-I.

CBT-I has many advantages for they avoid side effects of hypnotics. In addition, CBT use during pregnancy and breast feeding avoids the exposure of a fetus/newborn to medication. Also, many patients prefer CBT-I, avoiding difficulties in swallowing pills, and avoiding the concept of using a pharmacological approach. Unfortunately, CBT services are extremely limited (particularly amongst general practicioners) and costly. Efforts to increase delivery of CBT have been promising via self-help groups, computer and internet programs [16].

Thus, pharmacotherapy is the primary treatment for insomnia [1] with CBT being used in approximately 1% of chronic insomniacs [17]. Combining both treatments offers no major advantage acutely and only minimal advantage long-term [15].

In the 1970s, benzodiazepines came to the forefront in treating insomnia and are still widely prescribed. They provided a welcomed increase in safety profile over barbiturates (e.g., secobarbital and butalbital) and other barbiturate-like substances (e.g., ethchlorvynol and chloral hydrate). In particular, it was difficult to successfully overdose on them unless combined with other CNS suppressants such as alcohol [1,17,18]. Since many with insomnia have co-morbid psychiatric disorders, particularly depressive and anxiety disorders with suicidal behavior [5,6], suicide attempts with hypnotics frequently occur in this population. The dangers inherent with barbituates and barbiturate-like agents cannot be overemphasized. For example, chloral hydrate is a toxic dose at about five times the therapeutic dose and tolerance can develop after only days [19]. This is one of the main reasons why the usage of these drugs has been discouraged by sleep experts [1]; clearly, benzodiazepines are safer hypnotics. Although benzodiazepines have many positive safety features, they too have a number of undesirable effects. Most notably, benzodiazepines can induce dependence with subsequent withdrawal and rebound symptoms if they are suddenly discontinued. Furthermore, these drugs are subject to abuse, particularly in patients with a history of alcohol and drug abuse [1,17,18]. Since most of the approved benzodiazepines for insomnia have half-lives of over 8 hours (except for triazolam), it is not surprising that next-day (residual) fatigue, psychomotor and neuropsychological dysfunction are potential side effects; the longer the half-life of the drug, the more likely the occurrence of residual adverse events. There are five FDA approved benzodiazepines for insomnia (estazolam, flurazepam, quazepam, temazepam and triazolam) [20,21].

Triazolam, flurazepam, quazepam and estazolam are effective in sleep onset and sleep maintenance problems whereas temazepam is effective in only sleep-onset problems in adults, 18–65 years old; in adults 65 years and older, triazolam and flurazepam are helpful for sleep onset and maintenance problems, whereas temazepam has been shown to be helpful for only sleep maintenance problems [22].

In an attempt to improve the safety profile of benzodiazepines, the non-benzodiazepines (but also benzodiazepine receptor agonists) were developed in 1989 and FDA approved in 1993 [1,17,18,20,23,24,25,26,27]. Zolpidem was the first of these drugs developed; zolpidem as well as another nonbenzodiazepine, zaleplon, selectively attaches to the benzodiazepine recognition site on the GABA-A receptor at the level of the α-1 subunit while eszopiclone, also a non-benzodiazepine, is predominantly selective for the α-2 and α-3 subtypes with lesser sensitivity for α-1 subtype. In contrast, bezodiazepines have a more diffuse (non-selective) effect at the α subunit (α-1, α-2, α-3 and α-5 subtypes) [28,29]. This preferential selectivity by non-benzdiazepines and their short half-lives (8 h or less) are probably responsible for the suggested (but still debated) finding of a reduction in next-day fatigue as well as less psychomotor and neuropsychological dysfunction in comparison to benzodiazepines. Like benzodiazepines, these drugs are safe regarding overdoses. In contrast, dependence and withdrawal symptoms rarely develop with some believing that this is related to its specificity at the GABA-A receptor described above [30]. In addition, non-benzodiazepines tend not to be abused nor sought out by drug abusing populations in comparison to benzodiazepines. Thus, non-benzodiazepines are effective in insomnia and may offer a better safety profile over benzodiazepines. Nonetheless, they still share similar potential adverse events- sedation, anterograde amnesia, complex sleep-related behaviors, and impaired balance with subsequent falls. As suggested above, the superiority of non-benzodiazepines over benzodiazepines have recently been questioned [31]. Although the 2005 NIH consensus conference [1] and a recent meta-analysis [32] supported a superiority of nonbenzodiazepines over benzodiazepines, the reality is that there have been a minimal number of head to head studies. Recent reports suggest that that benzodiazepines and non-benzodiazepines have equivalent efficacy and side effect profiles leading NICE (The National Institute of Clinical Excellence) and other regulatory groups to support using benzodiazepines over non-benzodiazepines due to lower prices [31]. Currently there are seven FDA approved nonbenzodiazepines for insomnia which consist of zaleplon, eszopiclone, zolpidem and four derivative zolpidem preparations including zolpidem-extended release, zolpidem sublingual high dose (Edluar), zolpidem sublingual low dose (Intermezzo), and zolpidem oral spray (Zolpimist) (see Table 1 and Table 2) [24,25,26,27,33,34,35].

As referred to above, various zolpidem modifications recently have provided alternative delivery systems in order to increase the drug’s efficacy and target specific sleep disturbances (see Table 2). Zolpidem CR (6.25 and 12.5 mg dose) is a composite preparation which has a component that releases immediately and a component that is slowly released, supposedly allowing higher blood levels later in the sleep cycle (greater than for zolpidem IR) effecting sleep onset and sleep maintenance disturbances. This drug dissolves over an extended time with an increased half-life [27]. Unfortunately, there have been no head to head studies comparing zolpidem CR to zolpidem IR. Therefore, the superiority of the zolpidem CR preparation for sleep maintenance problems has not been definitively proven. In addition, two sublingual oral tablets (not to be swallowed) were developed- zolpidem SL (sublingual) (Edluar and Intermezzo) which dissolve in the mouth- as well as an oral spray (Zolpimist) [33,34,35] these latter three preparations apparently are more rapidly absorbed avoiding first pass effects of the liver, have shorter half-lives, and allow patients to not have to deal with swallowing a tablet or pill. Swallowing issues can be a problem, particularly in the elderly, a population representing the majority of insomniacs and hypnotic users [36]. The two sublingual preparations have been tweaked for different unique sleep disturbances. The sublingual preparation, Edluar was developed and FDA approved for sleep onset insomnia in 2009 [33]. The other sublingual preparation, Intermezzo was specifically developed and FDA approved (2011) for a subtype of sleep maintenance insomnia, *i.e.*, middle-of-the-night-wakefullness with difficulty returning to sleep. The dose is particularly small, 1.75–3.5 mg, and should be taken only if 4 h of bedtime remain prior to the time one must awaken [34]. The Zolpimist preparation is an oral spray preparation with each metered spray containing 5 mg of zolpidem. The drug was approved for sleep onset insomnia in 2008 [35].

In 2005, the FDA approved the first melatonin agonist, ramelteon (Rozerem), which is effective for sleep-onset problems (see Table 1). It has a short half-life (1–1.5 h). Its mechanism of action is as a melatonin receptor agonist acting via promoting drowsiness via MT1 receptor stimulation and synchronizing of the circadian clock via MT2 receptor stimulation. It has no effect on the benzodiazepine-GABA-A receptor [1,17,18,37]. Therefore, it appears to be free of possible drug abuse, dependence, and next-day cognitive dysfunction although one recent report suggests otherwise [38]. It is one of two prescribed hypnotics that is not a scheduled drug. In 2010, the FDA approved the first H_1_ antagonist and tricyclic antidepressant (TCA), doxepin (Silenor, 3 and 6 mg) for insomnia (see Table 1). At these small doses (in contrast, the antidepressant dose is 150–300 mg/day), doxepin (Silenor) was found to be effective for maintenance sleep problems without any significant side effects in both younger and older populations for periods up to 3–6 months. At these low doses, doxepin (Silenor) is preferentially a histaminic H_1_ receptor antagonist which is believed to be its main mechanism of action underlying its effect on insomnia [17,18,23,39,40]. Interestingly, doxepin (Silenor) is not effective for sleep-onset problems which is probably related to a long *T*_max_ of approximately 3.5 h (see Table 1) Although doxepin (Silenor) has minimal anti-cholinergic effects, out of caution, the PI states that it should be avoided in the presence of untreated narrow angle glaucoma or severe urinary retention [40]. Doxepin does not effect the GABA-A receptor and does not have any associated complications of abuse, tolerance, withdrawal, or complex sleep behavior. It is not a scheduled hypnotic, similar to ramelteon (all other hypnotics in Table 1 are schedule 1V drugs). Doxepin (Silenor) has minimal associated next-day residual effects (sedation, 6%–9% for Silenor and 4% for placebo) [40] which may relate to an early morning rise of histamine which is an “alerting neurotransmitter” [41]. Recently, Wu, Chang and Zu demonstrated that low dose doxepin (12.5 mg), the generic formulation, was effective in insomnia without inducing significant side effects (this study was not placebo-controlled) [42]. It may be that 10 mg doxepin capsules, the smallest generic capsule in the United States, would also be as effective and comparable to Silenor 3 or 6 mg. Comparative studies have not been performed.

**Table 1:** The Food and Drug Administration (FDA) approved hypnotics for insomnia.

Drug Class	Gerneric Name	Trade Name	Dose (mg)	Eliminations Half Life (h)	*T*_max_ (h)	Indication	DEA Class
Benzodiazepines	Estrazolam	Prosom^®^	1, 2	10–24	1.5–2	* SOI, SMI	IV
	Flurazepam	Dalmane^®^	15, 30	48–120	1.5–4.5	SOI, SMI	IV
	Quazepam	Doral^®^	7.5, 15	48–120	2–3	SOI, SMI	IV
	Temazepam	Restoril^®^	7.5, 15	8–22	1–2	SOI, SMI	IV
	Triazolam	Halcion^®^	0.125, 0.25	2–4	2–6	SOI	IV
Benzodiazepine Agonists	Zaleplon	Sonata^®^	5, 10, 20	1	1	SOI	IV
	Zolpidem	Ambien^®^	5, 10	2.5	1.6	SOI	IV
	Zolpidem CR	Ambien CR^®^	6.25, 12.5	2.8	1.5	SOI, SMI	IV
	Eszopiclone	Lunesta^®^	1, 2, 3	6	1	SOI, SMI	IV
Melatonin Agonist	Ramelteon	Rozerem^®^	8	1–2.6	0.75	SOI	None
H. Antagonist	Doxepin	Silenor^®^	3, 6	15.3	3.5	SMI	None
Orexin Antagonist	Suvorexant	Belsomra^®^	5–20	12	2	SOI, SMI	IV

* SOI (Sleep Onset Insomnia); SMI (Sleep Maintenance Insomnia); Resources used for data—see references [20,21,24,25,26,32,37,40,42].

**Table 2:** Zolpidem and its derivatives.

Generic Name	Trade Name	Dose (mg)	Elimination Half Life (h)	*T*_max_ (h)	Indication	DEA Class
Zolpidem	Ambien^®^	5, 10	2.5	1.6	* SOI	IV
Zolpidem SL	Edluar™	5, 10	2.9	1.4	SOI	IV
Zolpidem SL	Intermezzo^®^	1.75, 3.5	2.5	1.3	** SMI	IV
Zolpidem Oral Spray	Zolpimist^®^	5, 10	2.7	0.9	SOI	IV

* SOI Sleep Onset Insomnia; ** SMI Sleep Maintenance Insomnia characterized by middle-of-the-night awakening followed by difficulty returning to sleep. Resources used for data-see references [20,21,24,33,34,35].

In 2014, the FDA approved suvorexant (Belsomra), the first orexin receptor antagonist (for orexin 1 and orexin 2 receptor) for the treatment of insomnia with sleep onset and/or sleep maintenance difficulties (see Table 1); since orexin is a peptide that promotes wakefulness and effects the sleep wake cycle, suvorexant blocks these effects and induces sleep by being a dual orexin antagonist. It is approved for geriatric (≥65 years old) and non-geriatric populations. The dose range is 10–20 mg/day with a half-life of 12.2 h. In trials where suvorexant was given up to I year, there was no evidence of withdrawal, or rebound effects upon discontinuation of the medication. The main side effect was somnolence seen in 6.7% of patients on drug *vs*. 3% of patients on placebo. This side effect usually resolved with continued usage. Rarely, complex sleep-related behavior was observed for suvorexant (0.2%) *vs.* placebo (0%) [43,44,45]. Cataplexy was not observed in patients administered a therapeutic dose although it has occurred at higher doses; the latter is of theoretical interest since patients with narcolepsy are orexin deficient. Therefore, patients treated with a dual orexin antagonist might be expected to be associated with cases of cataplexy [43,44,45]. On a precautionary note, the PI does warn against administering suvorexant in patients with narcolepsy [45]. In general, suvorexant appears to have a more benign side effect profile than the benzodiazepines and non-benzodiazepines.

Clinicians frequently administer off-label medications for insomnia, particularly sedating antidepressants as a treatment of choice for insomnia (see Table 3) [1,17,18,46,47].

**Table 3:** Off-label medications for insomnia.

Drug Class	Gerneric Name	Trade Name	Dose (mg)	Eliminations Half Life (h)	*T*_max_ (h)
Sedating Antidepressants	Trazodone	Desyrel^®^ *	25–150	9 (7–15)	1–2
	Amitriptyline	Elavil^®^ *	10–100	30 (5–45)	2–5
Sedating Antipsychotics	Quetiapine	Seroquel^®^	25–100	6	1–2
Anihistamines	Diphenhydramine	Benadryl^®^	25–50	4–8	1–4
Anticonvusants	Gabapentin	Neurontin^®^	100–900	5–9	1.6–3

Resources used for data—see references [1,46,47]; * these 2 trade names are no longer available in USA.

Other than doxepin (Silenor), antidepressants are not FDA approved for insomnia. Sedating antidepressants such as trazodone and amitriptyline are not only routinely prescribed by clinicians but they are the most prescribed treatments for insomnia. The NIH State of Science Conference on insomnia treatments reviewed the status of antidepressants reporting minimal scientific evidence supporting their use [1]. Of particular concern in using antidepressants for insomnia are a number of unique potential side effects. TCA’s can have cardiovascular complications; they block α-1 adrenoceptors leading to orthostatic hypotension. They also slow intraventricular conduction and can lead to bundle-branch block and arrhythmias [48]. Trazodone, a heterocyclic antidepressant, has similar cardiovascular side effects as TCA’s [1,17,18,46,47]. Furthermore, trazodone is associated with a rare but serious side effect, priaprism, a sustained erection which can result in a urological emergency; this was recently reported after a single 100 mg dose [49], well within the dose range used for insomnia. Lastly, overdoses with sedating antidepressants, particularly TCA’s, (most have half-lives over 24 h) can lead to a successful suicide. For example, a 30 day prescription of 100 mg/day of amitriptyline (the dose range for insomnia ranges from 10–100 mg at bedtime) [18] falls within the lethal dose [50].

Other off-label hypnotics widely used by clinicians are sedating atypical antipsychotics, antihistamines, and anticonvulsants [1,17,18,46,47]. One of the most widely used is an atypical antipsychotic, quetiapine. Approved for schizophrenia and bipolar disorder, it is effective at lower doses for insomnia (25–100 mg/day). Unfortunately, it poses many medical risks including glucose dysregulation, tardive dyskinesia and an increased risk of stroke in those with dementia [18].

As recommended by the American Academy of Sleep Medicine (AASM) [51], off-label hypnotics may be considered in at least two situations: When FDA approved drugs are not efficacious for a particular patient (as many as 40% of insomniacs fail to respond) [15] and when an insomnia patient has a co-morbid condition that may actually benefit from this off-label drug which is FDA approved for the co-morbid condition. An example of the latter is in an insomnia patient with schizophrenia or bipolar disorder in which quetiapine might be tried or in a patient with a seizure disorder where anticonvulsants (e.g., gabapentin) might be appropriate, or an insomnia patient with severe allergies where antihistamines might be helpful.

## 2. Hypnotics in the Elderly

All of the FDA approved hypnotics have been found to be effective in the elderly. Dose reductions (starting with half the dose) are required for zolpidem, zaleplon and eszopiclone due to increased sensitivity in the elderly [1,17,18].

### 2.1. Short-Term vs. Long-Term

Prior to 2005, hypnotics, particularly benzodiazepines have been recommended for short-term use, 7–14 days, predominantly due to concerns of their side effects [1,17,18]. For example, benzodiazepines were only studied in short-term studies mainly focusing on a maximum of 2–4 weeks duration [22]. Unfortunately, insomnia frequently becomes chronic in many patients, lasting over 2 years [52]. Therefore, what are clinicians to do? The FDA has realized the need for long-term use of hypnotics as demonstrated by the fact that all hypnotics since 2005 have been studied long-term and approved with no limitation on duration of usage on the labeling [17,18]. Continued long-term efficacy and safety studies (both placebo-controlled, double-blind and open-label) ranging from 3 to 12 months have demonstrated that zolpidem, zaleplon, ezsopiclone, remelteon, doxepin, and suvorexant retain their efficacy without tolerance, abuse, withdrawal effects or any new adverse events developing [17,18,39,44,53,54,55,56,57]. Thus, clinicians may use hypnotics long-term if necessary; the need for continued use should be reassessed periodically.

If long-term use is necessary, one useful strategy is to administer hypnotics on an as needed basis-a few times a week *vs*. nightly. This would cut down on total medication exposure and minimize costs and possible side effects. This strategy was also based on the fact that insomnia frequently is not a nightly phenomenon. Interestingly, no matter whether medications were taken nightly or intermittently, there were no differences in efficacy, tolerance, or abuse [55].

### 2.2. Treatment Strategy Based on Specific Sleep Disturbances and Clinical Situations

Which sleep disturbance is actually present may help to determine which hypnotic medication to use. Patients with a sleep-onset disturbance might be best treated with a hypnotic with a short half-life (e.g., zaleplon, remelteon, triazolam, zolpidem IR, zolpidem oral spray, zolpidem sublingual-Edular); those with a sleep disturbance later in the evening or early in the morning might require a hypnotic that has a longer half-life (e.g., zolpidem ER, eszopiclone, temazepam, doxepin, or suvorexant). Patients who suffer from sleep awakenings in the middle of the night and cannot get back to sleep might best be treated with zolpidem sublingual (Intermezzo). Zaleplon (which is only FDA approved for sleep-onset difficulties) can also be used off-label for this indication since it has a very short half-life (1 h) and as long as the patient plans to remain in bed or be sleeping for another 4 h.

Unfortunately, insomniacs frequently have multiple sleep disturbances [58] and therefore, may need hypnotics with longer half-lives ranging from 2.5 to 8 h to treat both sleep onset and sleep maintenance problems (e.g., temazolam, zolpidem ER, eszopiclone, or suvorexant). Hypnotics with particular long half-lives in general should be avoided due to an increased likelihood of next-day sedation (e.g., flurazepam). One exception to avoiding longer acting hypnotics is when a patient with insomnia has significant daytime agitation and anxiety where daytime anxiolytic effects from the hypnotic might be helpful.

Clinical situations that guide clinicians to use one treatment *vs*. others are numerous. A patient may have severe pulmonary disease where CNS sedating medications should be avoided. Here, ramelteon or doxepin are favorable choices as well as a non-pharmacological approach-CBT-I. If pregnancy or breast feeding is foreseeable, pharmacotherapy should be avoided; CBT-I might be the treatment of choice. If there is a history of drug or alcohol problems, avoiding benzodiazepines and non-benzodiazepines and utilizing doxepin, remelteon or possibly CBT-I are recommended. Recently, ramelteon was used successfully in alcohol dependent insomniacs [59].

Insomnia patients with a co-morbid anxiety disorder may respond best to specific hypnotic treatments. For example, patients with insomnia who have co-morbid generalized anxiety disorder (GAD) appear to do well when eszopiclone is added to the anti-anxiety treatment of escitalopram; both insomnia and anxiety improved more significantly with the addition of eszopiclone *vs*. placebo [11]. Interestingly, when zolpidem was used in insomnia patients with GAD being treated with escitalopram, zolpidem (*vs*. placebo) had no added effect on reducing anxiety [10]. In addition, insomnia patients with GAD might be treated with benzodiazepines with or without a SSRI which might give relief of both insomnia and anxiety. Patients with insomnia co-morbid with PTSD appear to have a therapeutic response to both their insomnia and PTSD symptoms when treated with eszopiclone or an α-1 antagonist, prazosin as reported in double-blind, placebo-controlled studies [60,61]. The latter drug was particularly effective for PTSD nightmares [60]. Here again, zolpidem, when given to patients with insomnia co-morbid with PTSD, improved insomnia but PTSD symptoms failed to respond [62]. Furthermore, caution should be applied in administering benzodiazepine hypnotics in insomnia co-morbid with PTSD; they appear to be relatively ineffective for both insomnia and PTSD symptoms and may be abused in this vulnerable population which has a frequent co-morbid history of drug abuse) [60,63,64]; some investigators have even recommended that benzodiazepine use be contraindicated for this population [64].

Patients with insomnia and a co-morbid major depressive disorder (MDD) also seem to have preferential responses regarding their insomnia and mood disorder. Benzodiazepines when added to an antidepressant course are clearly helpful for insomnia. Evidence also suggests that depression may also be more responsive with the combination but the advantage may fade over time [65,66]. Eszopiclone (*vs*. placebo) when added to fluoxetine antidepressant treatment not only improved insomnia but also increased the speed and magnitude of the antidepressant response to fluoxetine [67]. When zolpidem was used as the hypnotic in another study of insomnia with co-morbid MDD being treated with the SSRI, escitalopram, zolpidem (*vs*. placebo) significantly improved insomnia without any additional effect on depression responsiveness [68]. The beneficial effect of eszopiclone on anxiety and depression in contrast to a negligible effect of zolpidem seen in the above studies in insomnia co-morbid with GAD and MDD is most interesting. It may be that the differences of these drugs on the GABA-A α subunits contribute to these specific findings; the specificity of eszopiclone for the α-2 and α-3 subtypes may contribute to anxiolytic and antidepressant properties as proposed in the animal literature [28,29,69].

## 3. New Concerns

As a result of anecdotal reports received by the Medwatch system that hypnotics occasionally caused “sleep-related complex behaviors”, the FDA in 2007 required a label change, warning of this for all hypnotics. These behaviors included “sleep driving”, “sleep-walking”, “sleep-eating” and “sleep-violence” occurring in the middle of the night without any memory of the activity [70].

Recently, the FDA required pharmacokinetic studies on various preparations of zolpidem after receiving complaints of carry-over effects the next morning involving driving and sedation; significantly high blood levels 8 h after ingesting zolpidem were found, particularly in women (15% of women and 3% of men). The FDA (2013) mandated a lowered starting dose (half the usual dose) in women and suggested a similar lower dose for men (many men will respond to a lower dose); they recommended similar caution for all hypnotic medications [71].

Some epidemiological studies have suggested that the use of hypnotic medications were associated with increased mortality [72,73]. Interestingly, this was recently confirmed in another study but after controlling for baseline characteristics and lifestyle issues, there was no longer an association of hypnotic use and increased mortality [74]. Since this potential association is so alarming, further studies must prospectively examine this.

## 4. Conclusions

Chronic insomnia is a prevalent disorder that must be treated. Clinicians can use either pharmacotherapy or CBT-I. Pros and cons of each are discussed, as well as the use of off-label hypnotic medications.
